# *SlWRKY33* and *SlPUB23* Negatively Regulate *Rx4*-Mediated Field Resistance to Bacterial Spot Race T3 in Tomato

**DOI:** 10.3390/plants15121871

**Published:** 2026-06-16

**Authors:** Yun Hyeong Lee

**Affiliations:** 1 Department of Vegetable Science, College of Horticulture, China Agricultural University, Beijing 100193, China; dbsguddl5@naver.com; Tel.: +82-10-8467-7611; 2 Independent Researcher, Icheon 17418, Republic of Korea

**Keywords:** tomato, bacterial spot, *Xanthomonas perforans*, Rx4, SlWRKY33, SlPUB23, CRISPR/Cas9, field resistance, plant immunity

## Abstract

Bacterial spot, caused by *Xanthomonas* species, is a destructive tomato disease that reduces yield and fruit quality worldwide. The tomato resistance gene *Rx4* confers hypersensitive response and field resistance to race T3 of *Xanthomonas euvesicatoria* pv. *perforans*, but downstream components associated with *Rx4*-mediated field resistance remain unclear. Here, we compared the susceptible processing tomato line OH 88119 with its near-isogenic line Rx4-1806 after spray inoculation with the race T3 strain Xv829. Transcriptome profiling at 1, 6, and 72 h post-inoculation identified limited transcriptional differences at 1 and 6 h, but 2247 differentially expressed genes at 72 h, including 1712 genes downregulated in Rx4-1806. Enrichment analyses highlighted plant–pathogen interaction, plant hormone signal transduction, and MAPK signaling pathways. Among candidate defense-related genes, *SlWRKY33* was downregulated in Rx4-1806 and selected for functional validation. CRISPR/Cas9-mediated knockout of *SlWRKY33* enhanced resistance in both OH 88119 and Rx4-1806, whereas *SlWRKY33* overexpression increased infected leaf area and bacterial population. Knockout of *SlPUB23* enhanced resistance in Rx4-1806 but not in OH 88119. These results suggest that *SlWRKY33* and *SlPUB23* negatively regulate tomato field resistance to bacterial spot race T3, with *SlPUB23* functioning in an *Rx4*-dependent manner.

## 1. Introduction

Tomato (*Solanum lycopersicum* L.) is one of the most widely cultivated vegetable crops worldwide and is affected by numerous bacterial, fungal, and viral diseases [1]. Among these, bacterial spot is a major foliar and fruit disease that occurs in tomato- and pepper-producing regions worldwide [2,3]. Disease development is favored by warm and humid conditions, and symptoms include water-soaked lesions, necrotic leaf spots, defoliation, and fruit lesions that reduce marketability. Because chemical control can be inconsistent and may increase production costs and environmental concerns, host resistance remains one of the most effective and sustainable strategies for disease management [4].

Bacterial spot of tomato is caused by diverse Xanthomonas species and races. Race T3, associated with *Xanthomonas euvesicatoria* pv. *perforans* or *Xanthomonas perforans* depending on taxonomic treatment, has been a major target for resistance breeding [2,3]. Several resistant germplasm resources have been identified, including PI 128216, Hawaii 7981, and related wild or semi-wild tomato accessions [5]. The *Rx4* locus on chromosome 11 confers resistance to race T3 and is associated with both hypersensitive response and field resistance [6,7]. Although the molecular basis of *Rx4*-mediated hypersensitive resistance has been investigated, the regulatory components underlying *Rx4*-mediated field resistance are less well understood.

Plant immunity involves multiple layers of recognition and signaling. Pattern-triggered immunity is initiated by recognition of pathogen-associated molecular patterns at the cell surface, whereas effector-triggered immunity is activated when intracellular resistance proteins perceive pathogen effectors [8,9]. These immune responses require extensive transcriptional reprogramming and involve MAPK cascades, calcium signaling, hormone signaling, reactive oxygen species, transcription factors, and protein turnover [10,11]. Therefore, comparing transcriptomic responses between susceptible and resistant near-isogenic lines provides a useful strategy for identifying genes associated with resistance.

WRKY transcription factors are important regulators of plant immunity. Some WRKYs act as positive regulators of defense gene expression, whereas others can negatively regulate immune output or contribute to susceptibility depending on the pathogen system and genetic background [12,13]. Similarly, plant U-box E3 ubiquitin ligases participate in the regulation of immune signaling through protein ubiquitination and degradation. Arabidopsis PUB22, PUB23, and PUB24 have been reported as negative regulators of pattern-triggered immunity, suggesting that related PUB genes may also modulate defense responses in crop species [14,15].

In this study, we used the susceptible line OH 88119 and the *Rx4*-containing near-isogenic line Rx4-1806 to investigate field resistance to bacterial spot race T3. By integrating time-course RNA-seq with functional validation using CRISPR/Cas9-mediated knockout and overexpression lines, we identified *SlWRKY33* and *SlPUB23* as negative regulators of resistance. These findings provide insight into the regulatory network associated with *Rx4*-mediated field resistance and identify candidate targets for improving tomato bacterial spot resistance.

## 2. Results

### 2.1. Rx4-1806 Exhibits Reduced Disease Symptoms After Race T3 Inoculation

**Figure 1:**
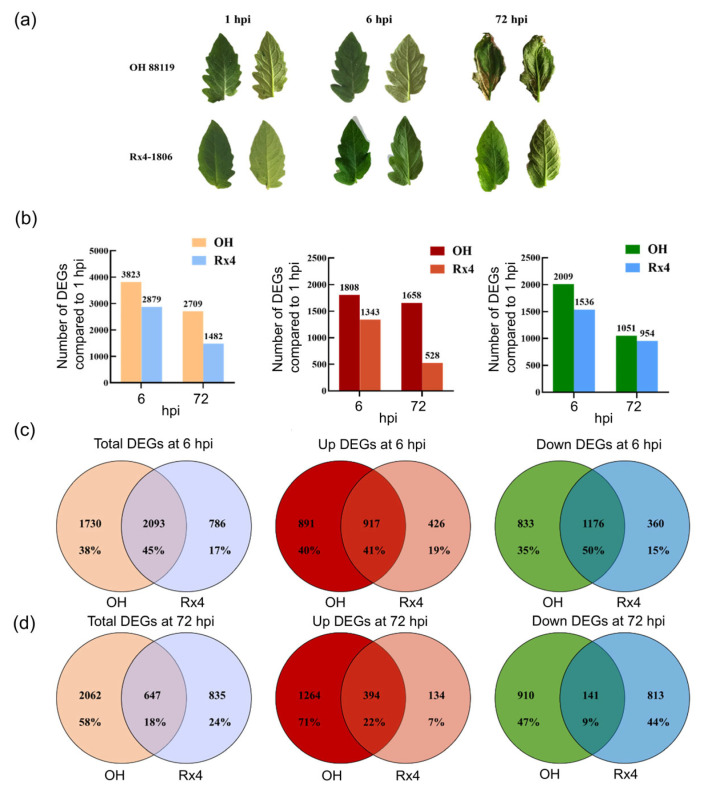
Phenotypic and transcriptomic responses of OH 88119 and Rx4-1806 after inoculation with race T3 strain Xv829. (**a**) Leaf symptoms at 1, 6, and 72 h post-inoculation (hpi). (**b**) Numbers of total, upregulated, and downregulated DEGs at 6 and 72 hpi compared with 1 hpi within each genotype. (**c**,**d**) Venn diagrams showing total, upregulated, and downregulated DEGs shared between or specific to OH 88119 and Rx4-1806 at 6 hpi (**c**) and 72 hpi (**d**).

OH 88119 is a processing tomato inbred line susceptible to bacterial spot race T3, whereas Rx4-1806 is a near-isogenic line carrying the resistance gene *Rx4* in the OH 88119 genetic background. To compare disease responses between the susceptible and resistant backgrounds, OH 88119 and Rx4-1806 plants were spray-inoculated with the race T3 strain Xv829 of *Xanthomonas euvesicatoria* pv. *perforans* (Figure 1a). At 1 and 6 h post-inoculation (hpi), leaves of both genotypes appeared phenotypically normal, with no visible lesions or necrosis. By 72 hpi, however, OH 88119 developed clear disease symptoms, including visible lesions and tissue necrosis, whereas Rx4-1806 showed only limited spot-like lesions. These phenotypic differences confirmed the enhanced resistance of Rx4-1806 to bacterial spot race T3. Although visible symptoms were not detected at 1 or 6 hpi, early host defense responses are expected to occur before macroscopic lesion development. Therefore, we selected these symptom-free early time points to capture initial infection-associated responses and 72 hpi to capture responses associated with visible disease progression and resistance expression. Among these time points, 1 hpi was used as the earliest post-inoculation reference point for within-genotype comparisons, allowing transcriptional changes at 6 and 72 hpi to be evaluated relative to the initial stage of infection.

### 2.2. Global Transcriptome Responses to Race T3 Inoculation

To investigate how OH 88119 and Rx4-1806 differ in their transcriptional responses to race T3 infection, we performed RNA-seq using leaf samples collected at 1, 6, and 72 hpi. Differentially expressed genes (DEGs) were identified using an absolute log2 fold-change cutoff of at least 1 and a false discovery rate (FDR) below 0.01. Because 1 hpi represented the earliest sampled stage after inoculation, it was used as the reference point for within-genotype comparisons at later time points. Because mock-inoculated samples were not included, these comparisons were interpreted as genotype-dependent temporal changes after inoculation rather than as absolute pathogen-induced responses relative to an untreated baseline.

Compared with 1 hpi, OH 88119 showed 3823 DEGs at 6 hpi and 2709 DEGs at 72 hpi, whereas Rx4-1806 showed 2879 and 1482 DEGs, respectively (Figure 1b). Thus, both genotypes underwent substantial transcriptional reprogramming after Xv829 inoculation, but the resistant Rx4-1806 line consistently exhibited fewer DEGs than the susceptible OH 88119 line. Venn diagram analysis further revealed genotype-dependent differences in infection-responsive genes. At 6 hpi, 2093 DEGs were shared between OH 88119 and Rx4-1806, accounting for 45% of total DEGs, whereas 1730 and 786 DEGs were specific to OH 88119 and Rx4-1806, respectively (Figure 1c). By 72 hpi, the number of shared DEGs decreased to 647, representing only 18% of total DEGs, while OH 88119-specific DEGs increased to 2062. Notably, Rx4-1806-specific downregulated DEGs increased from 360 at 6 hpi to 813 at 72 hpi (Figure 1d). These results suggest that OH 88119 and Rx4-1806 initially share a substantial portion of infection-responsive transcriptional changes, but their responses diverge markedly during disease progression. In particular, the accumulation of Rx4-1806-specific downregulated genes at 72 hpi suggests that suppression of selected host genes may be associated with *Rx4*-mediated resistance.

### 2.3. Rx4-Associated Transcriptional Differences Are Most Pronounced at 72 hpi

To identify genes associated with *Rx4*-mediated resistance, we next compared transcriptomes between Rx4-1806 and OH 88119 at each post-inoculation time point. Because Rx4-1806 is a near-isogenic line in the OH 88119 background, genotype-dependent transcriptional differences after Xv829 inoculation are expected to include genes associated with the *Rx4*-containing introgressed region and its downstream resistance response.

**Figure 2:**
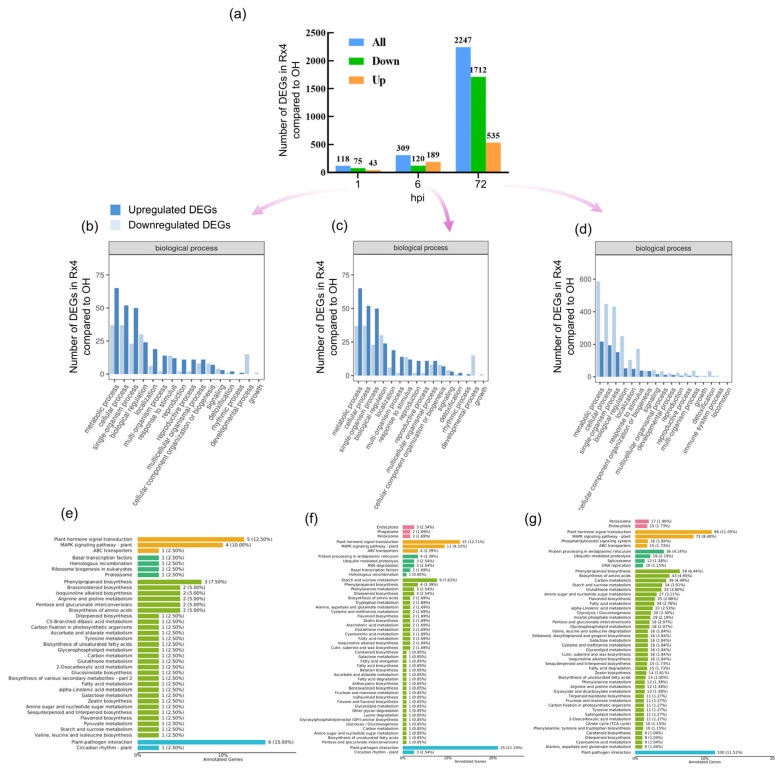
Functional classification of DEGs between Rx4-1806 and OH 88119 after race T3 inoculation. (**a**) Numbers of DEGs between Rx4-1806 and OH 88119 at 1, 6, and 72 hpi. (**b**–**d**) GO classification of upregulated and downregulated DEGs at 1 hpi (**b**), 6 hpi (**c**), and 72 hpi (**d**). (**e**–**g**) KEGG pathway classification of DEGs at 1 hpi (**e**), 6 hpi (**f**), and 72 hpi (**g**).

At 1 hpi, only 118 DEGs were detected between Rx4-1806 and OH 88119, including 43 upregulated and 75 downregulated genes in Rx4-1806 (Figure 2a). At 6 hpi, the number of DEGs increased modestly to 309, with 189 upregulated and 120 downregulated genes. By contrast, genotype-dependent transcriptional differences became much more pronounced at 72 hpi, when 2247 DEGs were detected. Among these, 1712 genes were downregulated, and 535 were upregulated in Rx4-1806 relative to OH 88119, indicating that the late transcriptional response associated with *Rx4*-mediated resistance was dominated by gene repression. Together with the within-genotype analysis, these results suggest that OH 88119 and Rx4-1806 share broadly similar early transcriptional responses to Xv829 infection, whereas their responses diverge substantially by 72 hpi, coinciding with visible disease development in OH 88119 and reduced symptom formation in Rx4-1806. The predominance of downregulated DEGs in Rx4-1806 at 72 hpi suggests that suppression of selected host genes may be an important component of *Rx4*-mediated field resistance to bacterial spot race T3.

### 2.4. Functional Classification of Rx4-Associated DEGs Highlights Defense-Related Pathways

To identify biological processes and pathways associated with *Rx4*-mediated resistance, we performed GO and KEGG enrichment analyses using DEGs detected between Rx4-1806 and OH 88119 at each time point after Xv829 inoculation. GO analysis showed that DEGs at 1, 6, and 72 hpi were commonly associated with broad biological processes, including metabolic process, cellular process, single-organism process, and biological regulation (Figure 2b–d). At 1 and 6 hpi, these categories included more upregulated than downregulated DEGs in Rx4-1806 relative to OH 88119. In contrast, by 72 hpi, downregulated DEGs became predominant across the major GO categories, consistent with the strong increase in repressed genes observed at this later time point.

KEGG analysis further revealed that several defense-related pathways were enriched among *Rx4*-associated DEGs. At 1 hpi, DEGs were already assigned to plant–pathogen interaction, plant hormone signal transduction, and MAPK signaling pathways, suggesting that genotype-dependent immune signaling begins shortly after inoculation (Figure 2e). At 6 hpi, these same pathways remained among the most represented categories, together with pathways related to endocytosis, phagosome, and peroxisome processes (Figure 2f). By 72 hpi, when OH 88119 showed clear disease symptoms, but Rx4-1806 remained less affected, the number of DEGs associated with defense-related pathways increased markedly. In particular, 100 DEGs were assigned to plant–pathogen interaction, 96 to plant hormone signal transduction, and 73 to MAPK signaling (Figure 2g). These results indicate that *Rx4*-mediated resistance is associated with transcriptional changes in core immune signaling pathways, with the largest pathway-level differences occurring at 72 hpi.

### 2.5. Identification and Expression Validation of SlWRKY33 as a Candidate Regulator of Rx4-Mediated Resistance

GO and KEGG analyses indicated that *Rx4*-associated DEGs were enriched in three major defense-related pathways: plant–pathogen interaction, plant hormone signal transduction, and MAPK signaling. Because genes represented in multiple defense pathways may have broader regulatory roles, we focused on DEGs assigned to two or more of these pathways and ranked them by expression change. This analysis identified ten candidate genes, including four genes associated with all three pathways and six genes associated with two pathways (Table 1). All ten candidates were assigned to the plant–pathogen interaction pathway, further supporting their potential involvement in the response to race T3 infection.

**Figure 3:**
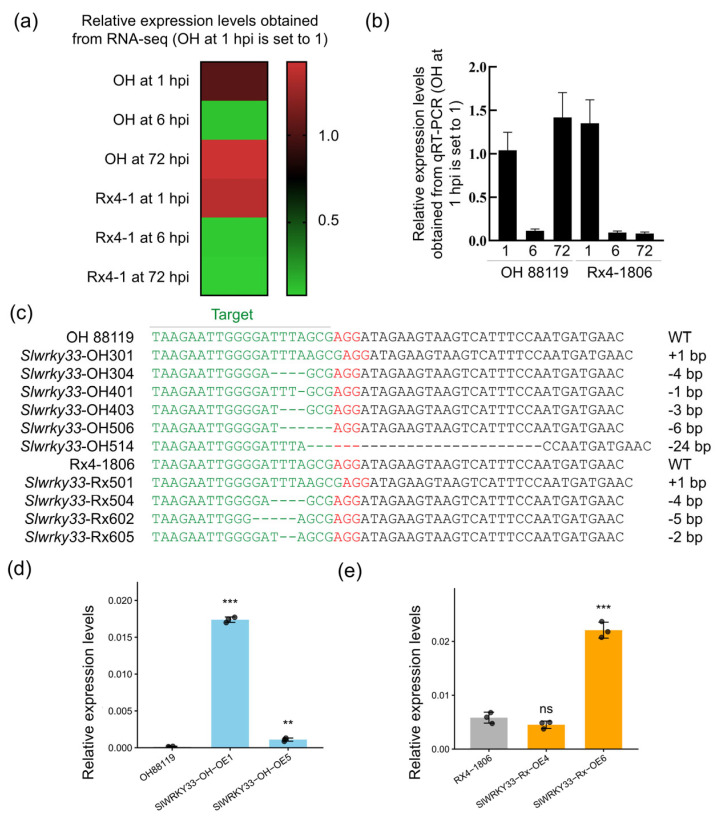
Identification of *SlWRKY33* as a candidate gene and generation of functional validation lines. (**a**) Relative *SlWRKY33* expression levels from RNA-seq, with OH 88119 at 1 hpi set to 1. (**b**) qRT-PCR validation of *SlWRKY33* expression after Xv829 inoculation. Error bars indicate SD from three independent biological replicates (n = 3). (**c**) CRISPR/Cas9-edited *SlWRKY33* alleles obtained in the OH 88119 and Rx4-1806 backgrounds. Target and PAM sequences are indicated. (**d**,**e**) Relative *SlWRKY33* expression levels in overexpression lines generated in the OH 88119 (**d**) and Rx4-1806 (**e**) backgrounds. Error bars indicate SD from three independent biological replicates (n = 3). Individual points represent biological replicates. Asterisks indicate significant differences compared with the corresponding parental control, OH 88119 in panel (**d**) and Rx4-1806 in panel (**e**), as determined by one-way ANOVA followed by Dunnett’s test. ** *p* < 0.01; *** *p* < 0.001; ns, not significant.

Among these candidates, only one gene, *Solyc09g007020*, annotated as a pathogenesis-related protein precursor, was upregulated in Rx4-1806 relative to OH 88119. The remaining nine candidates were downregulated in Rx4-1806. These included genes encoding LRR or receptor-like proteins, a calcium-binding protein, respiratory burst oxidase homolog protein B, and two WRKY transcription factors, Solyc06g066370 and Solyc09g014990, annotated as SlWRKY31 and SlWRKY33, respectively. Among these candidates, *SlWRKY33* was prioritized for functional characterization based on a combination of biological and expression-based criteria rather than fold change alone. *SlWRKY33* was assigned to both MAPK signaling and plant–pathogen interaction pathways, belonged to the WRKY transcription factor family with well-established roles in immune transcriptional regulation, and showed strong repression in Rx4-1806 after Xv829 inoculation (Figure 3a and Table 1). Its expression pattern was further supported by qRT-PCR validation (Figure 3b). We note that other candidates, including SlWRKY31 and respiratory burst oxidase homolog protein B, are also biologically relevant and may contribute to *Rx4*-associated resistance; however, their functional characterization was beyond the scope of this study.

To validate the RNA-seq results, we examined SlWRKY33 expression by qRT-PCR (Figure 3b). In the RNA-seq data, *SlWRKY33* showed a transient decrease followed by increased expression in OH 88119, whereas its expression remained reduced in Rx4-1806 after inoculation. qRT-PCR analysis showed a similar overall trend. Relative to OH 88119 at 1 hpi, *SlWRKY33* expression in OH 88119 decreased at 6 hpi and increased at 72 hpi. In contrast, *SlWRKY33* expression remained strongly reduced in Rx4-1806 at both 6 and 72 hpi. Although the magnitude of expression change differed slightly between RNA-seq and qRT-PCR, the overall expression patterns were consistent, supporting the reliability of the transcriptome data and suggesting that suppression of *SlWRKY33* is associated with the resistant response of Rx4-1806.

### 2.6. Generation of SlWRKY33 Loss-of-Function and Overexpression Lines

To determine whether *SlWRKY33* contributes to race T3 resistance, we generated *SlWRKY33* loss-of-function lines using CRISPR/Cas9 in both OH 88119 and Rx4-1806 backgrounds. Multiple independent edited lines were obtained in each genotype. In the OH 88119 background, the edited alleles included a 1 bp insertion and deletions ranging from 1 to 24 bp. In the Rx4-1806 background, the edited alleles included a 1 bp insertion and deletions ranging from 2 to 5 bp (Figure 3c). Homozygous T1 plants lacking the *Cas9* transgene were selected for subsequent disease-resistance assays.

Because *SlWRKY33* expression was reduced in Rx4-1806 after Xv829 inoculation, we also generated *SlWRKY33* overexpression lines to test whether increased *SlWRKY33* expression would alter disease resistance. In the OH 88119 background, two independent overexpression lines, *SlWRKY33*-OH-OE1 and *SlWRKY33*-OH-OE5, showed strong upregulation of *SlWRKY33* expression compared with the wild type (Figure 3d). In the Rx4-1806 background, *SlWRKY33*-Rx-OE6 showed increased *SlWRKY33* expression, whereas *SlWRKY33*-Rx-OE4 did not show clear overexpression and was therefore used cautiously in subsequent phenotypic interpretation (Figure 3e). These edited and overexpression lines provided genetic materials to test the functional role of *SlWRKY33* in bacterial spot race T3 resistance.

### 2.7. SlWRKY33 Negatively Regulates Resistance to Bacterial Spot Race T3

**Figure 4:**
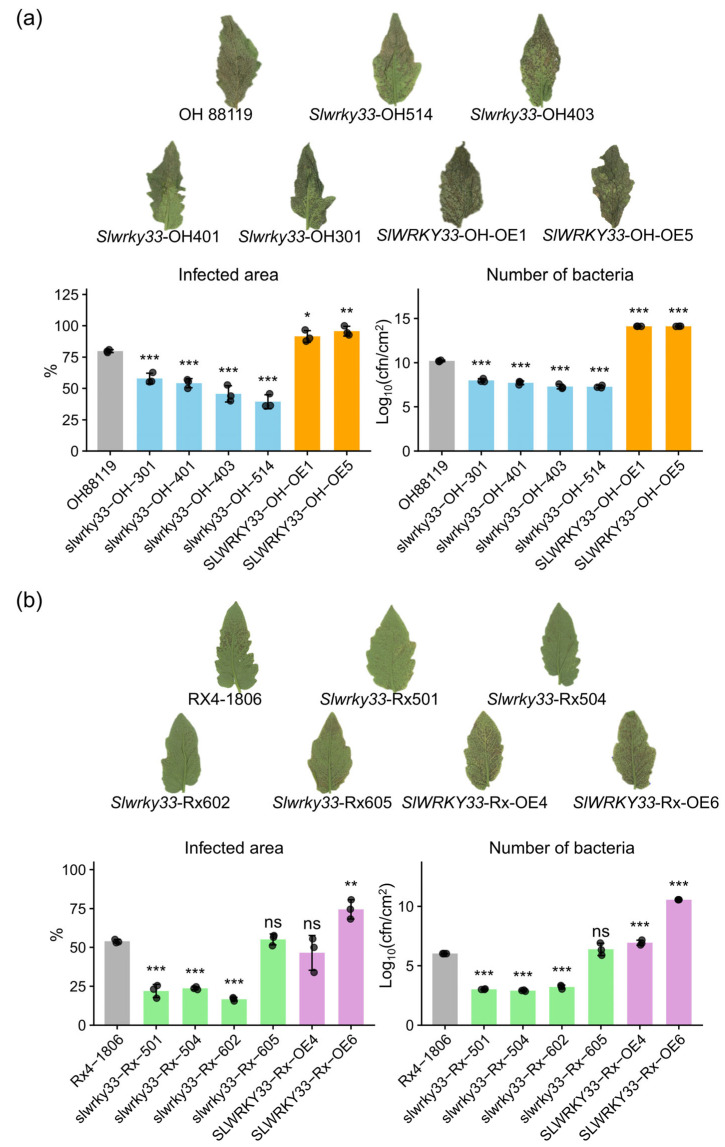
*SlWRKY33* negatively regulates resistance to bacterial spot race T3. (**a**) Disease symptoms, infected leaf area, and bacterial populations in OH 88119, *Slwrky33* knockout lines, and *SlWRKY33* overexpression lines after inoculation with Xv829. (**b**) Disease symptoms, infected leaf area, and bacterial populations in Rx4-1806, *Slwrky33* knockout lines, and *SlWRKY33* overexpression lines after Xv829 inoculation. Infected leaf area and bacterial population were quantified from independent biological replicates. Error bars indicate SD (n = 3). Individual points represent biological replicates. Asterisks indicate significant differences compared with the corresponding parental control, OH 88119 in panel (**a**) and Rx4-1806 in panel (**b**), as determined by one-way ANOVA followed by Dunnett’s test. * *p* < 0.05; ** *p* < 0.01; *** *p* < 0.001; ns, not significant.

To determine the function of SlWRKY33 in resistance to bacterial spot race T3, *SlWRKY33* loss-of-function and overexpression lines were spray-inoculated with Xv829 and evaluated by measuring infected leaf area and bacterial population. In the OH 88119 background, *Slwrky33* knockout lines showed reduced disease symptoms compared with OH 88119, whereas *SlWRKY33* overexpression lines showed more severe disease symptoms (Figure 4a). Quantification of infected leaf area confirmed that all tested *Slwrky33* knockout lines had significantly smaller lesion areas than OH 88119, while both overexpression lines had significantly larger lesion areas. Consistently, bacterial populations were significantly lower in the knockout lines and significantly higher in the overexpression lines than in OH 88119 (Figure 4a).

A similar pattern was observed in the Rx4-1806 background. Three *Slwrky33* knockout lines, *Slwrky33*-Rx602, *Slwrky33*-Rx501, and *Slwrky33*-Rx504, showed significantly reduced infected leaf area and lower bacterial populations compared with Rx4-1806 (Figure 4b). In contrast, the *SlWRKY33*-Rx-OE6 overexpression line showed increased infected leaf area and bacterial population. The *Slwrky33*-Rx605 knockout line and *SlWRKY33*-Rx-OE4 line did not differ significantly from Rx4-1806, consistent with their weaker or unclear functional alteration.

Together, these results show that loss of *SlWRKY33* enhances resistance to race T3, whereas increased *SlWRKY33* expression compromises resistance. Therefore, *SlWRKY33* functions as a negative regulator of tomato resistance to bacterial spot race T3 in both OH 88119 and Rx4-1806 genetic backgrounds.

### 2.8. SlPUB23 Negatively Regulates Race T3 Resistance in an Rx4-Dependent Manner

**Figure 5:**
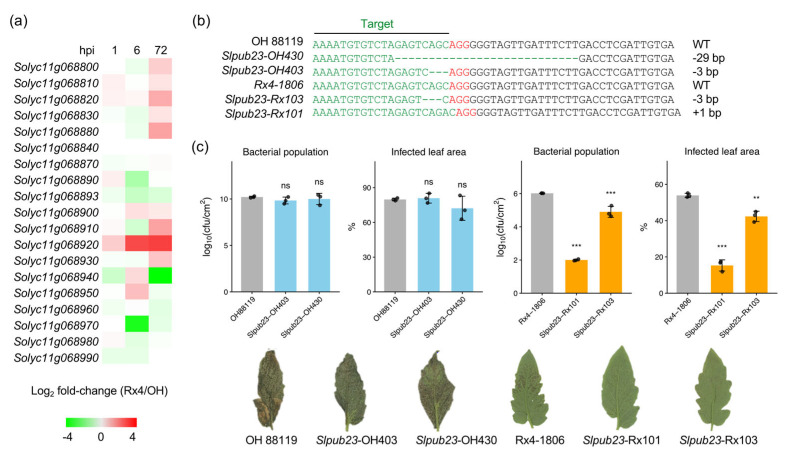
*SlPUB23* negatively regulates race T3 resistance in an *Rx4*-dependent manner. (**a**) Expression changes in genes located near the chromosome 11 resistance-associated region in Rx4-1806 relative to OH 88119 after Xv829 inoculation. The color scale indicates log_2_ fold change (Rx4-1806/OH 88119) at 1, 6, and 72 hpi. *Solyc11g068920*, corresponding to *SlPUB23*, is included among the genes shown. (**b**) CRISPR/Cas9-edited *SlPUB23* alleles generated in the OH 88119 and Rx4-1806 backgrounds. Target and PAM sequences are indicated. (**c**) Disease symptoms, infected leaf area, and bacterial populations in OH 88119, Rx4-1806, and corresponding *Slpub23* knockout lines after Xv829 inoculation. Infected leaf area was quantified from leaves collected 8–10 days after inoculation, and bacterial populations were measured from leaf disks collected 8 days after inoculation. Error bars indicate SD from three independent biological replicates (n = 3). Individual points represent biological replicates. Asterisks indicate significant differences compared with the corresponding parental control, OH 88119 or Rx4-1806, as determined by one-way ANOVA followed by Dunnett’s test. ** *p* < 0.01; *** *p* < 0.001; ns, not significant.

Because *SlPUB24*, located on chromosome 11, was previously implicated in tomato resistance to bacterial spot race T3 [16], we examined the expression patterns of nearby genes to identify additional candidates associated with *Rx4*-mediated resistance. Among the genes in this region, *Solyc11g068920*, annotated as *SlPUB23*, showed increased expression in Rx4-1806 relative to OH 88119 after Xv829 inoculation, particularly at 6 and 72 hpi (Figure 5a). Plant U-box E3 ubiquitin ligases regulate plant immunity by modulating the stability or activity of immune signaling proteins. For example, Arabidopsis PUB22, PUB23, and PUB24 negatively regulate PAMP-triggered immunity, and the nearby tomato U-box gene *SlPUB24* was previously shown to enhance resistance to *Xanthomonas euvesicatoria* pv. *perforans* race T3. Therefore, *SlPUB23* was selected for functional characterization as a candidate regulator of *Rx4*-associated resistance.

To test the function of *SlPUB23*, CRISPR/Cas9-edited lines were generated in both OH 88119 and Rx4-1806 backgrounds. In OH 88119, two edited lines were obtained: *Slpub23*-OH430, carrying a 29 bp deletion, and *Slpub23*-OH403, carrying a 3 bp deletion. In Rx4-1806, two edited lines were obtained: *Slpub23*-Rx103, carrying a 3 bp deletion, and *Slpub23*-Rx101, carrying a 1 bp insertion (Figure 5b).

After spray inoculation with Xv829, *Slpub23* mutants in the OH 88119 background showed disease symptoms comparable to those of OH 88119. Consistently, neither infected leaf area nor bacterial population differed significantly between OH 88119 and the *Slpub23*-OH mutant lines (Figure 5c). In contrast, *Slpub23* mutants in the Rx4-1806 background showed enhanced resistance. Both *Slpub23*-Rx103 and *Slpub23*-Rx101 had significantly reduced infected leaf area compared with Rx4-1806, and bacterial populations were also significantly lower in the mutant lines (Figure 5c). These results indicate that loss of *SlPUB23* enhances resistance only in the *Rx4*-containing background. Thus, *SlPUB23* acts as a negative regulator of bacterial spot race T3 resistance in an *Rx4*-dependent manner.

## 3. Discussion

Bacterial spot is a major constraint to tomato production, and host resistance remains one of the most effective strategies for disease management. The resistance gene *Rx4* confers both hypersensitive response and field resistance to race T3 of *Xanthomonas euvesicatoria* pv. *perforans*, but the downstream components associated with *Rx4*-mediated field resistance remain poorly understood. In this study, we used the susceptible line OH 88119 and its near-isogenic line Rx4-1806 to identify transcriptional and functional components associated with resistance to race T3.

RNA-seq analysis showed that transcriptional differences between OH 88119 and Rx4-1806 were limited at 1 and 6 hpi but became pronounced at 72 hpi, when disease symptoms were clearly visible in OH 88119 but remained limited in Rx4-1806. This late divergence may reflect cumulative differences in disease progression rather than a single early signaling event. At early time points, both genotypes were phenotypically symptomless, suggesting that pathogen perception and basal immune signaling may occur in both backgrounds. The genotype-differential DEGs detected at 1 and 6 hpi are therefore less likely to be secondary consequences of macroscopic tissue damage and may include early *Rx4*-associated immune responses. Consistent with this interpretation, KEGG classification detected genotype-differential genes assigned to plant–pathogen interaction, plant hormone signal transduction, and MAPK signaling pathways at 1 and 6 hpi. However, because mock-inoculated controls were not included, these early differences should still be interpreted as genotype-dependent post-inoculation responses rather than definitive pathogen-specific or *Rx4*-specific responses.

At 72 hpi, most DEGs in Rx4-1806 relative to OH 88119 were downregulated, suggesting that *Rx4*-mediated field resistance may involve suppression of selected host genes as well as activation of defense pathways. Similar patterns have been reported in previous transcriptome studies of tomato resistance to race T3, in which resistant materials showed extensive downregulation of DEGs compared with susceptible materials [17,18]. Repression may contribute to resistance through several non-mutually exclusive mechanisms. Genes highly expressed in susceptible OH 88119 at 72 hpi may include susceptibility-associated host factors, pathogen-responsive processes that support bacterial multiplication or lesion expansion, negative regulators of immunity, or stress- and damage-associated genes that are less activated in Rx4-1806 because of reduced disease progression. Thus, the large number of downregulated DEGs at 72 hpi may reflect both active suppression of susceptibility-associated pathways and reduced activation of disease-associated host responses.

GO and KEGG analyses indicated that *Rx4*-associated DEGs were enriched in plant–pathogen interaction, plant hormone signal transduction, and MAPK signaling pathways. These pathways are central to plant immunity, including pathogen recognition, immune signal transduction, and hormone-mediated defense regulation [8,9,10,11]. Reactive species may provide an additional layer connecting these pathways, because reactive oxygen and nitrogen species interact with MAPK cascades, hormone signaling, transcriptional regulation, and cell-death-associated responses during pathogen stress [19]. Therefore, the transcriptional divergence at 72 hpi may partly reflect differences in redox-associated immune signaling and tissue damage between OH 88119 and Rx4-1806.

Although our data link *Rx4*-mediated resistance with altered expression of *SlWRKY33* and an *Rx4*-dependent function of *SlPUB23*, they do not demonstrate that *Rx4* directly suppresses either gene. Rather, *Rx4* may influence these negative regulators indirectly through downstream immune signaling activated after Xv829 recognition. Because the transcriptome analysis was performed using bulk leaf tissue, the observed genotype-dependent transcriptional differences represent average responses across multiple cell types and cannot resolve whether *SlWRKY33* or *SlPUB23* function preferentially in specific cell populations. Recent advances in single-cell/nucleus RNA-seq and spatial transcriptomics have shown that plant stress responses can be highly cell-type-specific and spatially heterogeneous, and that such heterogeneity may be masked in bulk RNA-seq datasets [20].

Among candidate genes involved in multiple defense-related pathways, *SlWRKY33* was selected for functional validation because it was downregulated in Rx4-1806 and associated with both MAPK signaling and plant–pathogen interaction pathways. WRKY transcription factors are important regulators of plant immunity and can act as either positive or negative regulators depending on the pathosystem [12,13]. Consistent with this context dependence, tomato WRKY33 homologs can contribute positively to defense in some systems [21], whereas rice OsWRKY28 acts as a negative regulator of innate immunity [22]. In this study, *SlWRKY33* knockout reduced infected leaf area and bacterial populations in both OH 88119 and Rx4-1806, whereas *SlWRKY33* overexpression had the opposite effect. These reciprocal phenotypes indicate that *SlWRKY33* functions as a negative regulator of tomato resistance to bacterial spot race T3. However, the downstream immune genes regulated by SlWRKY33 remain unknown, and future chromatin-binding and transcriptome analyses will be needed to distinguish direct targets from indirect downstream effects.

We also characterized *SlPUB23*, a U-box E3 ubiquitin ligase gene located near *SlPUB24* on chromosome 11. The positional relationship between *SlPUB23* and *Rx4* is important for interpreting the *Rx4*-dependent phenotype of *Slpub23* mutants. Pei et al. fine-mapped *Rx4* to a 45.1 kb interval on chromosome 11 and identified an NBS-LRR gene as the candidate *Rx4* gene [7]. Subsequent reports have associated *Rx4*/Xv3 with *Solyc11g069020*, whereas *SlPUB23* corresponds to *Solyc11g068920* [23]. Thus, *SlPUB23* does not appear to be the fine-mapped Rx4 candidate gene itself, but is located in the same broader chromosome 11 resistance-associated region. The *Rx4*-dependent phenotype of *Slpub23* mutants may therefore reflect functional interaction with the *Rx4*-mediated immune pathway or tight regional linkage rather than identity with the *Rx4* locus.

Plant U-box proteins regulate immunity by modulating protein stability and immune signaling. Several PUB proteins, including Arabidopsis PUB22, PUB23, and PUB24, negatively regulate PAMP-triggered immunity, and PUB22 targets an exocyst subunit required for PAMP-triggered responses [14,15]. AcPUB23 in kiwifruit was also reported to negatively regulate immune responses to *Pseudomonas syringae* pv. *actinidiae* [24]. In our study, *SlPUB23* knockout did not alter disease resistance in OH 88119 but significantly reduced infected leaf area and bacterial populations in the Rx4-1806 background. These results suggest that *SlPUB23* functions within, or downstream of, the *Rx4*-associated resistance network rather than as a general regulator of basal resistance. One possible model is that SlPUB23 acts as a negative-feedback component that limits *Rx4*-mediated defense through ubiquitin-mediated turnover of positive immune regulators, such as receptor-complex-associated signaling proteins, trafficking-related proteins, or downstream components that sustain PTI/ETI amplitude. Direct SlPUB23 substrates remain to be identified.

Several limitations of the transcriptome analysis should be considered. First, RNA-seq data were analyzed using an FPKM-based differential expression pipeline. Although this approach was useful for initial candidate discovery, count-based methods such as DESeq2 or edgeR are now generally preferred because they model gene-level count distributions and better account for library composition. Because the GO and KEGG enrichment analyses were performed using DEG lists derived from this FPKM-based pipeline, the pathway enrichment results should also be interpreted cautiously as pathway-level indications for candidate prioritization rather than as definitive quantitative evidence of pathway activity. A full count-based reanalysis would likely generate a partially different DEG set and would require corresponding reanalysis of DEG summaries, Venn comparisons, GO/KEGG enrichment, candidate-gene prioritization, and pathway interpretation. Therefore, we interpret the transcriptome analysis primarily as a candidate-prioritization framework, while the main functional conclusions are supported by independent qRT-PCR validation and genetic assays using CRISPR/Cas9-edited and overexpression lines. Second, as noted above, mock-inoculated controls were not included, and the 1 hpi samples represent the earliest post-inoculation reference point rather than a pre-infection or mock-treated baseline.

The large group of genes downregulated in Rx4-1806 at 72 hpi remains mechanistically unresolved and may contain functionally distinct classes of genes. Some may represent susceptibility-associated host factors or pathogen-responsive genes activated during disease progression in OH 88119, whereas others may encode negative regulators of immunity whose repression contributes to resistance. Lower expression of some stress- and damage-associated genes in Rx4-1806 may also reflect reduced bacterial proliferation and tissue damage rather than direct transcriptional repression by *Rx4*. Therefore, our interpretation of this gene set should be viewed as hypothesis-generating. Future promoter-motif enrichment analysis of the 1712 downregulated genes, including tests for WRKY-binding W-box motifs and other hormone-, MAPK-, and redox-responsive cis-elements, would help determine whether coordinated repression during *Rx4*-mediated field resistance is associated with shared *cis*-regulatory features.

**Figure 6:**
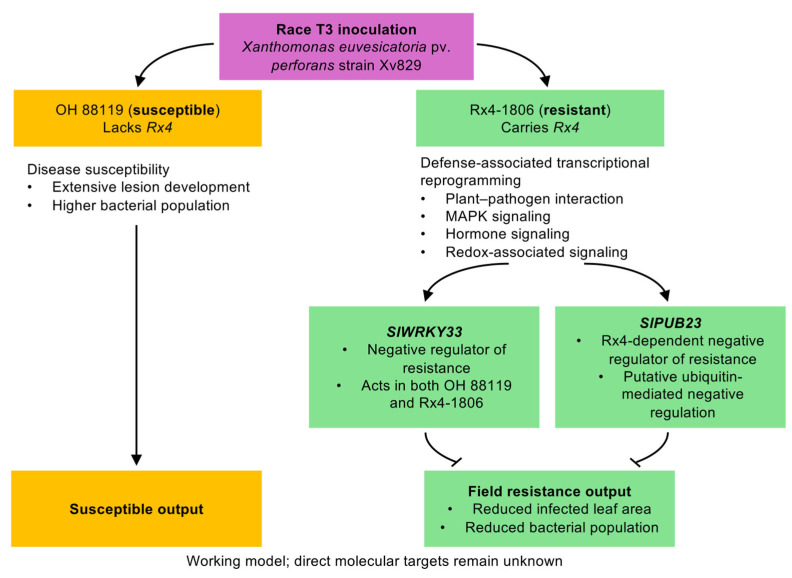
**Proposed working model of Rx4-mediated field resistance to bacterial spot race T3 in tomato**. Following inoculation with the race T3 strain Xv829 of *Xanthomonas euvesicatoria* pv. *perforans*, the susceptible line OH 88119, which lacks *Rx4*, develops disease susceptibility characterized by extensive lesion development and higher bacterial population. In contrast, the *Rx4*-containing near-isogenic line Rx4-1806 shows defense-associated transcriptional reprogramming involving plant–pathogen interaction, MAPK signaling, hormone signaling, and redox-associated signaling pathways. Functional analyses indicate that *SlWRKY33* acts as a negative regulator of resistance in both OH 88119 and Rx4-1806, whereas *SlPUB23* acts as an *Rx4*-dependent negative regulator, potentially through ubiquitin-mediated regulation. Suppression or disruption of these negative regulators is associated with enhanced field resistance, including reduced infected leaf area and reduced bacterial population. This model summarizes the current interpretation of the data; direct molecular targets remain unknown.

Together, these findings support a working model in which *Rx4*-mediated field resistance involves defense-related transcriptional reprogramming and suppression of negative regulatory components (Figure 6). *SlWRKY33* appears to act as a negative regulator of race T3 resistance in both OH 88119 and Rx4-1806, whereas *SlPUB23* functions specifically in the *Rx4*-containing background. However, this study tested only the race T3 strain Xv829 under controlled conditions. Future studies should evaluate *Slwrky33* and *Slpub23* mutants across additional tomato backgrounds, *Xanthomonas* races or strains, unrelated pathogens, and field environments, and should determine whether their loss affects plant growth, fruit traits, or other agronomic traits.

## 4. Materials and Methods

### 4.1. Plant Materials and Pathogen Strain

The susceptible tomato inbred line OH 88119 and the near-isogenic line Rx4-1806 were used. Rx4-1806 was developed in the OH 88119 genetic background and carries the bacterial spot race T3 resistance gene *Rx4*. The race T3 strain Xv829 of *Xanthomonas euvesicatoria* pv. *perforans* was used for pathogen inoculation. *Escherichia coli* DH5α and *Agrobacterium tumefaciens* GV3101 were used for plasmid propagation and tomato transformation, respectively.

### 4.2. Plant Growth Conditions

Tomato seedlings were grown in 128-cell trays containing peat moss, vermiculite, and perlite at a 2:1:1 ratio. Seedlings approximately 10 cm tall were transferred to 9 cm pots and maintained at approximately 25 °C under a 16 h light/8 h dark photoperiod.

### 4.3. Bacterial Culture and Inoculation

The Xv829 strain was recovered from −80 °C stocks on yeast extract–dextrose–calcium carbonate (YDC) medium and incubated at 28 °C for 48–72 h. Bacteria were resuspended in sterile water containing 10 mM MgSO_4_ and adjusted to OD600 = 0.22–0.25. Silwet L-77 was added at 250 μL/L. Tomato plants with five to six true leaves were spray-inoculated with the bacterial suspension. Inoculations were performed using independent biological replicates as described for RNA-seq sampling and disease-resistance assays below. To promote disease development, plants were maintained under warm and humid conditions by covering with plastic film and misting leaves regularly after inoculation. Mock-inoculated controls were not included in the RNA-seq experiment; therefore, the transcriptome data were interpreted as genotype-dependent temporal changes after inoculation, with 1 hpi used as the earliest post-inoculation reference point.

### 4.4. RNA-Seq Sampling, Library Preparation, and Sequencing

For transcriptome analysis, OH 88119 and Rx4-1806 plants were inoculated with Xv829. Leaf samples were collected at 1, 6, and 72 hpi. For each genotype and time point, five plants were pooled as one biological replicate, and three independent biological replicates were collected. Total RNA was extracted using a plant RNA extraction kit, and RNA quality and concentration were assessed with a NanoDrop 2000 spectrophotometer. RNA-seq library construction and sequencing were performed by Beijing Biomarker Technologies Co., Ltd., (Beijing, China).

### 4.5. RNA-Seq Data Processing and Differential Expression Analysis

Raw reads were filtered to obtain clean reads and aligned to the tomato reference genome ITAG4.0 using HISAT2 v2.0.4 [25]. Transcripts were assembled using StringTie v2.2.1 [26]. Gene annotation was performed using the KEGG Ortholog, NCBI non-redundant protein, and Gene Ontology databases. Gene expression was normalized as FPKM. DEGs were identified using an absolute log2 fold change of at least 1 and FDR < 0.01. GO enrichment analysis was performed with GOseq [27], and KEGG enrichment analysis was performed with KOBAS [28,29]. Transcription factor prediction was conducted using iTAK [30]. Heatmaps were generated using pheatmap after Z-score standardization of expression values.

### 4.6. qRT-PCR Analysis

Reverse transcription was performed using HiScript II Q RT SuperMix for qPCR. RT-qPCR was performed using ChamQ SYBR qPCR Master Mix in a 10 μL reaction. *EF-1α* (*Solyc06g0050600*) was used as the internal reference gene. Relative expression was calculated using the 2^−ΔΔCt^ method [31]. Primers used for RT-qPCR are listed in Table S1.

### 4.7. Construction of CRISPR/Cas9 and Overexpression Vectors

CRISPR/Cas9 vectors were constructed to target *SlWRKY33* (*Solyc09g014990*) and *SlPUB23* (*Solyc11g068920*). Target sgRNAs were designed using CRISPR-P 2.0. The CRISPR/Cas9 system consisted of CP041/pHSE401-tomatoU6 and CP043/pCBC-DT1T2-tomatoU6. CP043/pCBC-DT1T2-tomatoU6 was used as an intermediate sgRNA cassette vector, and CP041/pHSE401-tomatoU6 was used as the binary CRISPR/Cas9 vector for tomato transformation. In this system, sgRNA expression was driven by the tomato U6 promoter. The CP041/pHSE401-tomatoU6 binary vector contains the kanamycin resistance (*nptII*) selectabl/e marker for plant transformation, and transformed tomato tissues were selected on regeneration medium containing kanamycin at 100 mg/L. The assembled constructs were introduced into *Escherichia coli* DH5α for plasmid propagation and sequence verification. Verified plasmids were transformed into *Agrobacterium tumefaciens* GV3101, and positive transformants were selected on LB medium containing kanamycin and rifampin. For *SlWRKY33* overexpression, the coding sequence of *SlWRKY33* was cloned into the Super1300-cFlag binary vector. All constructs were verified by PCR and sequencing before tomato transformation.

### 4.8. Tomato Transformation and Identification of Transgenic Lines

Stable tomato transformation was performed using an Agrobacterium-mediated cotyledon transformation system based on a previously described method [32]. Briefly, cotyledon explants from OH 88119 and Rx4-1806 were infected with Agrobacterium tumefaciens GV3101 carrying the corresponding CRISPR/Cas9 or overexpression construct, regenerated on selective medium, and transferred to rooting medium before transplantation. Regenerated transgenic plants were obtained from both genetic backgrounds, although regeneration efficiency differed between OH 88119 and Rx4-1806. For gene-edited plants, genomic DNA was extracted from T0 and T1 plants, and PCR products flanking the target sites were sequenced. Homozygous T1 plants lacking *Cas9* were used for disease assays. For overexpression lines, vector integration was confirmed by PCR, and increased *SlWRKY33* expression was verified by RT-qPCR.

### 4.9. Disease Resistance Assays

For phenotypic evaluation, plants were spray-inoculated with Xv829 as described above. For infected leaf area measurements, diseased leaves were collected 8–10 days after inoculation. Five symptomatic leaves were collected from each plant, scanned at 600 dpi, and analyzed using ASSESS v2.2. The average percentage of infected leaf area from five leaves was used as the value for each plant [33]. For bacterial population measurements, leaf disks were collected 8 days after inoculation. For each genotype, leaf disks were collected from inoculated plants, ground in sterile water, serially diluted, plated on YDC medium, and incubated at 28 °C for 48 h. Colony counts were calculated from plates containing 30–400 colonies and expressed as log10 (cfu/cm^2^). The number of biological replicates used for infected leaf area and bacterial population measurements is indicated in the corresponding figure legends.

### 4.10. Statistical Analysis

Statistical analyses were performed in R. For experiments in which multiple edited or overexpression lines were compared with the corresponding parental control, statistical significance was determined using one-way ANOVA followed by Dunnett’s multiple-comparison test. OH 88119 or Rx4-1806 was used as the parental control, depending on the genetic background. This analysis was applied to the qRT-PCR data for *SlWRKY33* overexpression lines in Figure 3d,e and to the infected leaf area and bacterial population data in Figure 4 and Figure 5. Data are presented as mean ± SD. Significance was defined as *p* < 0.05, *p* < 0.01, or *p* < 0.001, as indicated in the figure legends.

## 5. Conclusions

This study investigated transcriptional and functional components associated with *Rx4*-mediated field resistance to bacterial spot race T3 in tomato. Comparative transcriptome analysis of OH 88119 and Rx4-1806 identified large genotype-dependent transcriptional differences at 72 hpi, with defense-related pathways including plant–pathogen interaction, hormone signal transduction, and MAPK signaling enriched among *Rx4*-associated DEGs. Functional analyses showed that *SlWRKY33* negatively regulates resistance in both OH 88119 and Rx4-1806, whereas *SlPUB23* negatively regulates resistance specifically in the *Rx4*-containing background. These findings suggest that suppression of negative regulatory components contributes to *Rx4*-mediated field resistance. However, the breeding value of *SlWRKY33* and *SlPUB23* as candidate targets for improving bacterial spot resistance should be further validated across additional tomato genetic backgrounds, pathogen strains, and field environments.

## Figures and Tables

**Table 1:** Candidate DEGs associated with multiple defense-related pathways in Rx4-1806 compared with OH 88119 after race T3 inoculation.

Gene ID	Annotation	Expression Change * in Rx4-1806 vs. OH 88119	Defense-Related Pathway(s)
*Solyc09g007020*	Pathogenesis-related protein precursor	2.4	MAPK signaling; plant hormone signal transduction; plant–pathogen interaction
*Solyc01g009810*	LRR protein 1-like	−2.6	MAPK signaling; plant hormone signal transduction; plant–pathogen interaction
*Solyc07g066550*	LRR receptor-like protein	−2.7	MAPK signaling; plant hormone signal transduction; plant–pathogen interaction
*Solyc02g076830*	Epidermis-specific secreted glycoprotein EP1-like	−2.8	MAPK signaling; plant–pathogen interaction
*Solyc03g083470*	Rust resistance kinase Lr10-like protein	−3.2	MAPK signaling; plant hormone signal transduction; plant–pathogen interaction
*Solyc06g066370*	WRKY transcription factor 31	−3.3	MAPK signaling; plant–pathogen interaction
*Solyc02g088090*	Calcium-binding protein CML30	−3.5	MAPK signaling; plant–pathogen interaction
*Solyc07g055400*	G-type lectin S-receptor-like serine/threonine-protein kinase	−3.7	MAPK signaling; plant hormone signal transduction; plant–pathogen interaction
*Solyc09g014990*	WRKY transcription factor 33	−4.1	MAPK signaling; plant–pathogen interaction
*Solyc01g099620*	Respiratory burst oxidase homolog protein B	−4.4	MAPK signaling; plant–pathogen interaction

* Positive and negative values indicate higher and lower expression, respectively, in Rx4-1806 relative to OH 88119. LRR, leucine-rich repeat; MAPK, mitogen-activated protein kinase.

## Data Availability

The raw RNA-seq datasets generated and analyzed in this study have been deposited in the NCBI Sequence Read Archive (SRA) under BioProject accession number PRJNA1459031. Other data supporting the findings of this study are available from the corresponding author upon reasonable request.

## Supplementary Materials

The following supporting information can be downloaded at: https://www.mdpi.com/article/10.3390/plants15121871/s1, Table S1: Primers used in this study; Figure S1: Gene structures and editing target sites of *SlWRKY33* and *SlPUB23*.

## Abbreviations

The following abbreviations are used in this manuscript:

DEG	Differentially expressed gene
ETI	Effector-triggered immunity
FDR	False discovery rate
GO	Gene Ontology
hpi	Hours post-inoculation
KEGG	Kyoto Encyclopedia of Genes and Genomes
MAPK	Mitogen-activated protein kinase
PTI	Pattern-triggered immunity
qRT-PCR	Quantitative reverse-transcription PCR
