# Supplementary material for: SlWRKY33 and SlPUB23 Negatively Regulate Rx4-Mediated Field Resistance to Bacterial Spot Race T3 in Tomato
Source: Plants (Basel). 2026 Jun 16;15(12):1871. doi: 10.3390/plants15121871 (PMC13306445; doi:10.3390/plants15121871)
Supplement: Supplementary file 1 [file plants-15-01871-s001.zip › Table S1.pdf]

Table S1. Primers used in this study.

Primer name	Sequence (5′–3′)	Purpose
YZPUB23-2-F	AGGGGTAGTTGATTTCTTGAC	RT-qPCR for *SlPUB23*
YZPUB23-2-R	TTGTTCGCGATTGAACATGA	RT-qPCR for *SlPUB23*
EF-1αRT-F	TACTGGTGGTTTTGAAGCTG	RT-qPCR internal reference, *EF-1α*
EF-1αRT-R	AACTTCCTTCACGATTTCATCATA	RT-qPCR internal reference, *EF-1α*
CrisprPUB-F	ATATATGGTCTCGTTTGTTTCTATGTCCAATCT CAAGTTTTAGAGCTAGAAATAGC	CRISPR/Cas9 editing of *SlPUB23*
CrisprPUB-R	ATTATTGGTCTCGAAACCTGCTGACTCTAGAC ACATCCAAACTACACTGTTAGATTC	CRISPR/Cas9 editing of *SlPUB23*
Crispr-WRKY33	ATATATGGTCTCGTTTGAAGAATTGGGGATTT AGCGGTTTTAGAGCTAGAAATAGC	CRISPR/Cas9 editing of *SlWRKY33*
Crispr-WRKY33-1	ATTATTGGTCTCGAAACGTAGATGGACTTAAT GAATCCAAACTACACTGTTAGATTC	CRISPR/Cas9 editing of *SlWRKY33*
FLAG-WRKY33-F	ATACACCAAATCGACTCTAGAATGGCTTCTTC AGGTGGAAATATGA	*SlWRKY33* overexpression construct
FLAG-WRKY33-R	ACTAGTATTTAAATGTCGACCGTTAAGGAAA GAGCTGAAGAATAAA	*SlWRKY33* overexpression construct
IFPUB23-3-F	CTTCTTCACATTCACACAC	Identification of *SlPUB23* edited lines
IFPUB23-R	CCAAGAATTGATTATCATTATGC	Identification of *SlPUB23* edited lines
T1PUB23-3-F	TAGATTGCCTTAGGAGGATCA	Genotyping of *SlPUB23* T1 edited lines
T1PUB23-3-R	ATATTCAAAGCTTCATCACTCG	Genotyping of *SlPUB23* T1 edited lines
IFQCWR-7-F	ATGGCTTCTTCAGGTGGAAATATGA	Identification of *SlWRKY33* edited lines
IFQCWR-4-R	TTCAGAATTGCCCTCTTTA	Identification of *SlWRKY33* edited lines
T1QCWR-1-F	CTTCTTCGTTTAGCGATCTTC	Genotyping of *SlWRKY33* T1 edited lines
T1QCWR-1-R	CAAGATAAGAGGAAGGCGA	Genotyping of *SlWRKY33* T1 edited lines
SNAP-WRKY33-F	ATGGCTTCTTCAGGTGGAAATATGA	Identification of *SlWRKY33* overexpression lines
superNR	CAAGACCGGCAACAGGATTCAATCT	Identification of *SlWRKY33* overexpression lines
CP-JC1	ACGACGGCCAGTGCCAAG	Sequencing primer
CPJC3-2F	AATCCCACTATCCTTCGCAAGACCC	Sequencing primer
CPJC3-2R	GACCACCTCCTCGAAGTTCCAC	Sequencing primer
superCF	GACGCCATTTCGCCTTTTCAG	Sequencing primer
