# Supplementary figures and images for: SlWRKY33 and SlPUB23 Negatively Regulate Rx4-Mediated Field Resistance to Bacterial Spot Race T3 in Tomato
Source: Plants (Basel). 2026 Jun 16;15(12):1871. doi: 10.3390/plants15121871 (PMC13306445; doi:10.3390/plants15121871)

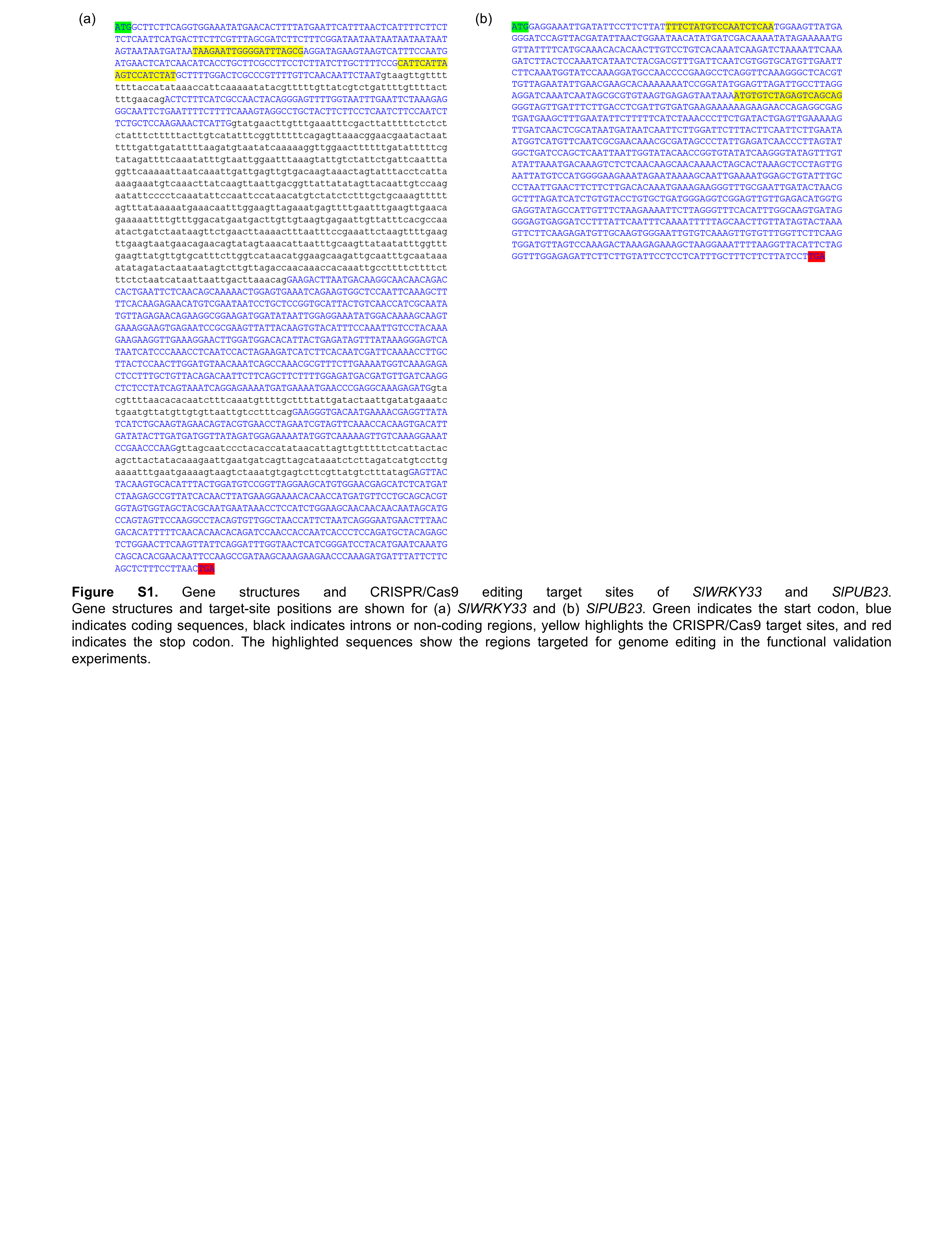

Supplement: Supplementary file 1 [file plants-15-01871-s001.zip › Figure S1_v1.png]
